# Proposed Physiological Mechanisms Underlying the Association between Adverse Childhood Experiences and Mental Health Conditions: A Narrative Review

**DOI:** 10.3390/children11091112

**Published:** 2024-09-12

**Authors:** Stefan Kurbatfinski, Aliyah Dosani, Deborah M. Dewey, Nicole Letourneau

**Affiliations:** 1 Department of Community Health Sciences, Cumming School of Medicine, University of Calgary, Calgary, AB T2N 1N4, Canada; stefan.kurbatfinski@ucalgary.ca (S.K.); adosani@mtroyal.ca (A.D.); dmdewey@ucalgary.ca (D.M.D.); 2 Owerko Centre, Alberta Children's Hospital Research Institute, Calgary, AB T2N 1N4, Canada; 3 Faculty of Health, Community and Education, School of Nursing and Midwifery, Mount Royal University, Calgary, AB T3E 6K6, Canada; 4 O’Brien Institute for Public Health, Cumming School of Medicine, University of Calgary, Calgary, AB T2N 1N4, Canada; 5 Departments of Pediatrics, Cumming School of Medicine, University of Calgary, Calgary, AB T2N 1N4, Canada; 6 Hotchkiss Brain Institute, University of Calgary, Calgary, AB T2N 1N4, Canada; 7 Faculty of Nursing, University of Calgary, Calgary, AB T2N 1N4, Canada; 8 Departments of Psychiatry, Cumming School of Medicine, University of Calgary, Calgary, AB T2N 1N4, Canada

**Keywords:** adverse childhood experiences, mental health, mechanisms, physiology, inflammation, epigenetics

## Abstract

Adverse childhood experiences (ACEs; e.g., physical abuse) can impact lifelong mental health both directly and intergenerationally, with effects transmitted from the parent to the child. Several physiological mechanisms have been proposed to explain the impacts of ACEs on mental health. The purpose of this narrative review was to synthesize and critique the peer-reviewed literature on physiological mechanisms proposed to underlie the impacts of ACEs on mental health, specifically: (1) hypothalamic–pituitary–adrenal axis functioning, (2) inflammation, (3) genetic inheritance and differential susceptibility, (4) epigenetics, (5) brain structure and function, (6) oxidative stress, and (7) metabolic profiles. We searched Google Scholar using variations of the terms “adverse childhood experiences”, “mechanisms”, and “mental health” to locate relevant peer-reviewed literature. We also mined citations of the identified literature to find additional important sources. The role of inflammation in the etiology of mental health conditions among those exposed to ACEs appeared promising, followed by hypothalamic–pituitary–adrenal axis functioning, brain structure and function, genetics, epigenetics, metabolism, and lastly, oxidative stress. Replication studies that examine the associations among ACEs, genetic inheritance and differential susceptibility, epigenetics, oxidative stress, and metabolism are required to better define links with mental health.

## 1. Introduction

Adverse childhood experiences (ACEs), defined as adverse events experienced before 18 years of age such as abuse, neglect, or poor living conditions, are associated with mental health conditions that can persist throughout childhood and into adulthood [1]. However, the pathways through which ACEs exert their influence are not entirely clear [1]. Several physiological mechanisms have been proposed through which ACEs may impact mental health. These include, but are not limited to, the hypothalamic–pituitary–adrenal (HPA) axis [2], inflammation [3], genetic inheritance and differential susceptibility [4], epigenetics [5], brain structure and function [6], oxidative stress [7], and metabolic profiles [8]. These physiological mechanisms are important for healthy human functioning and may serve to increase the risk of or resiliency to developing mental health conditions [2,3,5]. Moreover, the impact of ACEs on mental health through these physiological mechanisms can occur if: (1) the child or adolescent directly experiences an ACE and/or (2) the effects of ACEs experienced by caregivers are intergenerationally transmitted to the child or adolescent pre- or postnatally. Therefore, it is important to consider the timing of exposure when examining the impacts of ACEs on mental health [5,9]. A greater understanding of the physiological mechanisms that underlie the associations between ACEs and mental health conditions may enable researchers, policymakers, government bodies, and healthcare providers to better support individuals exposed to ACEs and prevent or alleviate mental health conditions.

A plethora of studies point to a dose–response relationship between the number of ACEs one has experienced and the risk of developing mental health conditions [1]. First observed by Felitti et al., having four or more ACEs was associated with higher odds of developing alcohol use disorder, depression, and attempting death by suicide compared to having zero ACEs, after controlling for sociodemographic variables [10]. In fact, in their meta-analysis of 37 studies, Hughes et al. observed a 37.48-fold higher odds of attempting death by suicide among individuals who experienced four ACEs compared to those who experienced no ACEs [1]. These findings continue to be replicated across numerous systematic reviews, which was captured in an umbrella review [11]. The type of ACE exposure is also important to consider, as certain types of ACEs have been more strongly associated with specific mental health conditions (e.g., emotional abuse has been more strongly associated with depression relative to physical abuse) [12]. Since considerable evidence points to a positive association between ACEs and various mental health conditions [1], understanding the physiological mechanisms through which ACEs impact mental health could help in developing interventions that reduce mental health impacts.

From a population health perspective, certain groups (e.g., racialized people) experience more ACEs due to systems of oppression, which in turn increases their risk of developing mental health conditions [13,14]. For example, Madigan et al. reported a higher prevalence of ACEs among certain racial minority groups (e.g., Indigenous peoples, Latinx individuals), low-to-moderate income families, and those experiencing homelessness in their meta-analysis of 206 studies [15]. Consistent with these findings, McCabe et al. in a nationally representative American cross-sectional study reported that sexual and gender minority groups reported more ACEs than their heterosexual counterparts alongside comorbid substance use and mental health challenges [16]. When considering the effects of ACEs on mental health outcomes, one must consider the interactions among determinants of health and various psychosocial factors across specific groups and populations.

## 2. Purpose of the Review and Methods

This review applied a narrative synthesis approach to examine and critique the existing literature, including studies and reviews, on physiological mechanisms proposed to increase the risk of and resilience to mental health problems in the context of ACEs. Narrative synthesis is a structured, but non-systematic approach to the synthesis of evidence on a topic; it is not bound by specific inclusion or exclusion criteria, and therefore, the number of included and excluded studies are usually not provided [17,18]. Relevant mechanisms were identified through discussion with academic experts (co-authors AD, DD, and NL) and via an examination of the important literature. The seven mechanisms identified above were examined in this review. Because nearly all described mechanisms are interrelated and are influenced by one another, we briefly touch on this when warranted; however, further discussion of this is beyond the scope of the paper.

To identify the relevant literature, one author (SK) searched through the existing literature, using different variations of the terms “adverse childhood experiences”, “mechanisms”, and/or “mental health problems”, alongside each respective physiological mechanism in Google Scholar. SK also mined identified articles and reviews for additional relevant articles. Studies were evaluated according to the hierarchy of evidence to determine the strength of evidence for a given mechanism [19]. Specifically, studies were ranked from most to least strong in terms of evidence as outlined by the hierarchy of evidence, ranging from systematic reviews and meta-analyses, randomized controlled trials, cohort studies, case-control studies, cross-sectional study designs, and lastly, to animal models [19]. However, for the purpose of this review, animal models could be particularly important in explaining physiological mechanisms as conditions and exposures among animals can be manipulated in a controlled manner [19]. While applying the hierarchy of evidence to critique the identified literature, we also purposefully considered the quantity of available evidence for each type of study design. For example, if 10 prospective cohorts suggested a link between two variables, but a randomized controlled trial did not, the findings of prospective cohort studies were considered as outweighing the results of the randomized controlled trial.

## 3. Hypothalamic–Pituitary–Adrenal (HPA) Axis Functioning

Individuals who experience ACEs are more likely to reside in conditions of, or be exposed to, toxic stressors (e.g., poverty and physical neglect) [20]. Toxic stress is described as a form of chronic stress that results in hyperactivation of the HPA axis [10]. The HPA axis governs the stress response system of many animals, including humans, via stress molecule signaling (e.g., cortisol) and adaptive functioning (e.g., enhancing sensory systems to better adapt to a threat) [21]. Under acute or tolerable levels of stress, the HPA axis assists in successfully navigating the stressors to return to homeostatic conditions more rapidly [21]. However, when exposed to toxic forms of stress, such as ACEs, the HPA axis can become hyperactivated, resulting in cortisol dysregulation and stress sensitization [20]. Consequently, a dysregulated HPA axis can negatively alter physiological processes (e.g., metabolism), increase one’s likelihood of engaging in risky health behaviors (e.g., smoking), and change brain architecture and function [20], all of which can increase the risk of developing mental health conditions [22]. The HPA axis is therefore important to examine as a mechanism that may increase the risk of or resilience to developing mental health conditions after ACE exposure, both prenatally and postnatally.

### 3.1. Impacts of ACEs on Individuals’ HPA Axis Functioning

Much evidence supports an association between exposure to ACEs and HPA axis dysregulation, though findings regarding the direction of the association are conflicting [23]. However, it appears that ACEs most often lead to a state of hypocortisolism in the absence of mental health conditions [23] or to blunted cortisol responses, particularly among women, when stressors are experimentally introduced [24,25]. It is thought that this state of hypocortisolism is acquired over time as the body becomes sensitized to toxic stressors (e.g., ACEs), whereby individuals release less cortisol in response to stressors due to a higher threshold for experiencing stress [23]. For women, greater estrogen levels may play a role in observed blunted cortisol responses when an external stressor is introduced [24,25]. Through this mechanism, ACEs may initially result in cortisol hypersecretion; however, the negative feedback mechanism of cortisol results in eventual hyposecretion through an adaptive response [23]. In a randomized controlled trial, McLaughlin et al. compared cortisol levels in children randomly assigned to remain in poor-quality institutional care, children randomized to high-quality foster care from poor-quality institutional care, and children growing up in non-institutionalized care in more typical Romanian family environments [2]. Children who were randomized to high-quality foster care at a young age approximated cortisol levels of children growing up in non-institutionalized care, as opposed to children remaining in poor-quality institutional care, who demonstrated blunted cortisol levels [2]. This study provides initial evidence supporting a causal relationship between ACEs and hypocortisolism; however, it focused mainly on neglect as the type of ACE [2]. Although hypocortisolism may serve as an adaptive means of responding to stress among those who experienced ACEs, insufficient cortisol secretion can prevent adequate responses to future stressors and result in other health sequelae (e.g., hypotension, weight loss, fatigue) [26].

Despite meta-analytic evidence of blunted cortisol responses among those who experienced ACEs [24,27], HPA dysregulation appears to change in direction when individuals also report a mental health condition, such that states of hypercortisolism are often observed [23]. Findings from systematic reviews consistently suggest that hypercortisolism is associated with major depressive disorder, bipolar disorder, and schizophrenia, and other mental health disorders [23]. There is also evidence that different types of ACEs predict different types of mental health conditions [28], highlighting the importance of considering the type of ACE experienced. Nevertheless, blunted cortisol responses have still been observed among individuals with a mental health condition (e.g., major depressive disorders) in response to morning and afternoon stressors [23]. These differences may arise from differences in the type and severity of ACE experienced, individual differences (e.g., genetic predispositions to certain mental health conditions), the timing of the exposure, the approach to measuring cortisol (e.g., at baseline versus introducing a stressor), and other confounders [23]. For example, it appears that abusive ACEs compared to neglective ACEs are often associated with mood disorders and that hypocortisolism is observed more frequently among older individuals reporting mood disorders [23]. More consistency in study design among various investigations is needed to better understand how these factors interact within an already complex system of physiological processes.

### 3.2. Mothers’ Prenatal Stress Is Intergenerationally Associated with Individuals’ HPA Functioning

Hypercortisolism and cognitive deficiency in children have been linked to mothers’ prenatal stress and HPA axis dysfunction [5,9,14,29,30]. This may be reflective of the fetal programming phenomenon, which posits that fetal development is affected by prenatal conditions to promote the child’s survival in its anticipated postnatal conditions [5]. Rat and human models show that highly stressed mothers are more likely to transmit cortisol via the placenta due to lower concentrations of the nβ-HSD2 enzyme [9]. The nβ-HSD2 enzyme is responsible for breaking down cortisol to its inactive form; in failing to moderate the level of cortisol that the fetus is exposed to, fetal HPA axis development is altered to function in anticipation of high stress conditions [5,9]. It appears that fetal programming mechanisms may be a proxy for the intergenerational transmission of a hyperactive HPA axis [5,14], predisposing children to developing anxious tendencies and mental health conditions through excessive levels of circulating cortisol [31]. This evidence suggests that the effects of ACEs could intergenerationally impact children’s mental health through the HPA axis as early as the prenatal period [5,9]. Therefore, to better understand how ACEs may impact mental health through the HPA axis, both prenatal and postnatal factors need to be considered.

### 3.3. Quality of Evidence

The evidence from systematic reviews, along with the described randomized controlled trial by McLaughlin et al. [2], provide considerable support for the notion that ACEs are associated with HPA axis dysregulation, with more evidence pointing to states of hypocortisolism in the absence of mental health conditions [23]. However, the associations between HPA axis functioning and mental health are often described as weak, which may decrease inferences of causality [23]. Further, when including psychopathology in such models or prenatal stress through fetal programming mechanisms, the evidence points to states of hypercortisolism among children and adults [23]. This suggests that hypercortisolism is linked more to mental health conditions among individuals exposed to ACEs. Conversely, hypocortisolism may be more linked to physical health conditions, such as hypotension and weight loss, which in turn may be indirectly associated with mental health problems [28]. Some studies conducted postnatally failed to account for prenatal factors, which could confound the association between ACEs, HPA axis functioning, and the development of mental health conditions [23]. It is crucial that more rigorous and consistent techniques are applied when collecting cortisol and data on the type, severity, frequency, and timing of ACEs exposures, as these factors are often attributed to account for the differences observed among studies that have examined ACEs, HPA functioning, and mental health outcomes. While there are discrepancies regarding whether states of hypo- or hypercortisolism are associated with mental health problems among those exposed to ACEs, a dysregulated cortisol response (regardless of direction) is associated with the etiology of mental health problems, revealing an important target for intervention.

## 4. Inflammation

Attention has also been given to the role of the immune system and associated inflammatory responses in the etiology of mental health conditions among those exposed to ACEs [3]. The immune system plays a critical role in sensing and responding to stress, impacting neural architecture and subsequent developmental trajectories [3]. It is composed of two processes: (1) the innate immune process, which uses physical barriers and creates an inflammatory response (e.g., swelling, skin color change) that provides a more rapid response and (2) the acquired immune process, which uses immune cells to create proteins (e.g., antibodies) that can specifically bind to antigen threats [3]. Typically, the former is deactivated once the biological threat is eliminated (inflammation is reduced), whereas antigen-specific antibodies produced in the latter continue to circulate throughout the body even after the threat is eliminated in case the body is exposed to the same threat [3]. A healthy immune system orchestrates these two processes without compromising healthy cells, whereas a dysregulated immune system harms healthy cells and causes adverse effects on health [3]. Toxic stressors, such as ACEs, have been associated with poorer immune system functioning and more inflammation, leading to more infections and allergies [3,32]. Therefore, the immune system is important to consider when examining the development of mental health conditions among individuals exposed to ACEs.

Proinflammatory cytokines are chemical messengers involved in governing the innate and acquired immune systems and play an important role in development and health [3]. Cytokines are also the molecules that cause sickness symptoms (e.g., fevers) [3]. There is evidence to suggest that proinflammatory cytokines (e.g., C-reactive proteins) play a role in the etiology of mental health conditions among children and adults through their effects on the human body [3]. Proinflammatory cytokines activate the HPA axis, whereby the subsequent release of cortisol inhibits the HPA axis and inflammatory response through a negative feedback mechanism [3]. An overabundance of cortisol results in a weakened inflammatory response while inadequate levels of cortisol result in an overactive inflammatory response [3]. Therefore, individuals with hypocortisolism are more likely to have an excess of proinflammatory cytokines circulating in their body, resulting in fatigue, lower motivation, and decreased physical activity, all of which increase the risk of developing mental health conditions such as depression [3,33]. While HPA axis functioning may appear to be a major contributor to mental health conditions, inflammation may underlie the effects and should be investigated in the context of ACEs and mental health.

### 4.1. Inflammation in the Context of the Prenatal Period

The prenatal period is particularly important to consider as a large portion of the immune system is developed during this time [5]. Prenatal stress has been observed to underlie dysregulated immune system responses (e.g., inflammatory responses) among infants through the placental transfer of immune-related molecules [5]. Prenatal stress and depression are more likely among mothers who have experienced ACEs [30] and have been associated with elevated levels of inflammatory cytokine markers during pregnancy, which can adversely affect fetal development through a surplus of inflammatory molecules [5]. Studies have also shown that depression before or during pregnancy is associated with higher levels of, as opposed to changes in, inflammatory markers throughout pregnancy [34,35]. The time at which pregnant mothers report experiencing stress or depression is also important, such that the first and third trimesters appear to be periods when mothers experience the most vulnerability to psychosocial risk factors (e.g., low social support; [5]). The relationship between prenatal stress and inflammation in children is supported through a systematic review, which revealed strong positive associations between prenatal stress and atopic diseases such as dermatitis which are triggered by an overproduction of certain inflammatory marker in childhood [33]. Since the fetal environment is instrumental in shaping the development of the infant’s immune system, adverse prenatal conditions can compromise healthy immune system functioning.

### 4.2. Inflammation in the Context of the Postnatal Period

Studies also demonstrate compelling evidence that direct experiences of ACEs result in dysregulated inflammatory responses [3]. In the early period following birth, infants who are physically neglected or malnourished are less likely to obtain certain immune-related molecules through breastmilk [3]. Experiences of ACEs past infancy have also been associated with heightened inflammatory responses [3]. For example, children in institutionalized care (56%) as opposed to non-institutionalized children (5%) have been found to have higher levels of antibodies for herpes- and meningitis-related antigens [3]. A systematic review of 11 studies conducted by Soares et al. demonstrated a strong and positive association between exposure to ACEs and elevated levels of inflammatory molecules such as proinflammatory cytokines, though some non-significant associations were also reported [36]. Similarly, Baumeister et al. observed significantly higher levels of C-reactive proteins and other proinflammatory cytokines among individuals exposed to childhood trauma in their meta-analysis of 25 studies [37]. Since an overly active immune system can increase the risk of developing poor mental health in the context of ACEs by producing symptoms that induce fatigue and lower motivation, which in turn, can lead to physical inactivity [33], the use of anti-inflammatory medication could help to reduce inflammatory cytokine levels and promote positive mental health outcomes, as evidenced in other studies [38].

### 4.3. COVID-19 and Inflammation: A Unique Interaction Exacerbating Mental Health Conditions

COVID-19 infection has been associated with greater inflammation, and for some individuals without an adequate immune response, infection with COVID-19 has resulted in cytokine storms [39,40]. For individuals with Long COVID, there are distinct patterns of inflammatory cytokine levels associated with greater levels of inflammation in the blood [39,41]. Long COVID has also been associated with various mental health conditions, such as depression, anxiety, suicidal ideation, and post-traumatic stress disorder [41]. It is possible that Long COVID impacts mental health conditions through the interaction between an inadequate immune response and cytokine release syndromes (e.g., cytokine storms), leading to the persistence of cytokine inflammatory molecules in the body [40,41]. With evidence that COVID-19 infection is associated with poorer physical health outcomes (e.g., hospitalization) for those with ACEs [42], alongside the compelling evidence that ACEs result in hyperactive immune systems [3,34,35], it is likely that individuals with ACEs who experienced a COVID-19 infection are at greater risk of developing an exaggerated inflammatory response and at increased risk of developing mental health conditions. Such an interaction is only proposed. Further investigation is needed to better understand the effects of COVID-19 on inflammation and whether it could have sequelae in relation to the other proposed physiological mechanisms discussed in this review.

### 4.4. Quality of Evidence

The evidence regarding the impacts of ACEs on inflammation and mental health, both prenatally and postnatally, is promising, suggesting that inflammatory mechanisms may underlie mental health conditions among individuals exposed to ACEs [33,36]. Not only do most studies suggest a positive association between ACEs and levels of immune-related molecules (which are associated with fatigue and depression), these associations are often established as strong in terms of their magnitude [33,36]. Additionally, studies, which have evaluated the effects of anti-inflammatory treatments, have reported a reduction in depressive symptoms [38]. There also appears to be consistency in how inflammatory biomarkers are collected, increasing the quality of the converging findings [33,36]. Nevertheless, consideration of and consistency in the type, timing, frequency, and severity of ACEs exposure would further strengthen the research literature and provide more clarity on how and when to best intervene. Investigating which proinflammatory molecules are most closely linked to specific mental health conditions would further help guide the development of effective mental health interventions.

## 5. Genetics

Children inherit deoxyribonucleic acid (DNA) from their parents, a molecule that contains the gene sequences specific to an individual [43,44]. Certain alleles (i.e., one of two versions of a genetic sequence in a particular region of a chromosome) in gene sequences are known to confer risk of developing mental health conditions [44]. Those who inherit certain functional alleles (i.e., alleles that affect the function of an encoded protein) from their parents may exhibit more regulated protein expression, optimizing physiological functioning and subsequently promoting mental health outcomes; conversely, other functional alleles that abnormally code for proteins usually place children at risk of health-related, educational, and relational difficulties, influencing their quality of life and overall mental health [44]. This suggests that some children may be predisposed to worsened mental health through a decreased genetic capacity to regulate their response to external stressors [43,45]; however, this is not always the case [4]. Research leading to the development of the differential susceptibility hypothesis suggests that certain genotypes may prove disadvantageous and even harmful when exposed to negative environmental conditions (e.g., ACEs), while the same genotype may reap the most benefits when exposed to positive environmental conditions [4]. This suggests that it is important to not only identify relevant genes to mental health conditions, but also how these genes function in diverse adverse (or non-adverse) conditions.

Although individuals with chromosomal disorders (e.g., in which individuals inherit too few or too many chromosomes such as Down syndrome) report higher prevalence of certain mental health conditions [46], we focus exclusively on studies that have examined the interaction between non-chromosomal disordered genotypes and ACEs to predict mental health conditions. Several genes have been established as important in the context of ACEs. We briefly introduce some key candidate genes/genetic sequences that are recognized as playing an instrumental role in mental health development in the context of ACEs (Table 1).

### 5.1. Genetic Resilience to Mental Health Conditions in the Context of ACEs

The serotonin transporter promoter polymorphism (5-HTTPLR) variant of the SLC6A4 gene has variant long (L) and short (s) alleles that affect the level of serotonin expression [47]. Serotonin is an important neurotransmitter primarily associated with regulating neurocognitive processes by directing when satiation of the reward system is achieved; the L allele of the 5-HTTPLR has historically been described as the more beneficial allele [47]. However, in a review that captured six studies on the role of the 5-HTTPLR gene and mental health resilience in the presence of adversity, three reported that the L/L genotype was protective while three reported that the s/s genotype was protective [52]. The oxytocin receptor gene (OXTR) plays an important role in stress regulation, promoting cognitive functioning, social relationships, and one’s self-esteem [50]. Two studies reported a risk effect for children with the OXTR G/G genotype when exposed to adversity [53,54], whereas another team reported protective effects of the G/G genotype [48]. Interestingly, another study revealed a significant interaction between mothers’ prenatal stress and infants’ OXTR genotype, such that infants with two or more risk genotypes (e.g., single-nucleotide polymorphisms Rs53576 GG and rs2254298 A) exhibited less self-regulation when exposed to prenatal stress; these findings support gene-by-environment interactions as early as the prenatal period [55]. However, there is also evidence that the Rs53576 GG genotype is associated with positive outcomes (e.g., increased trust behavior), supporting the differential susceptibility hypothesis whereby homozygosity of this genotype (G/G) may prove advantageous or disadvantageous in the absence or presence, respectively, of ACEs [56]. Lastly, the brain-derived neurotrophic factor (BDNF) gene has been identified as an important contributor to children’s resilience, playing an important role in neuroplasticity, neuronal growth and maintenance, and glucose utilization [51]. Three studies observed protective effects for those with the Val (Valine)/Val genotype as compared to those with other genotypes, such as the Met (Methionine) allele variation [52,57].

The corticotropin releasing hormone receptor 1 (CRHR1) is an important gene as it is a fundamental gene that helps govern the HPA axis system through its effect on cortisol production [48]. The TAT haplotype of the CRHR1 has been observed to be protective in the face of adversity based on two studies [48,58], while others show additional risk effects based on other genetic groups such as those with the GGT haplotype or the G allele [58,59]. Other genes, such as the FK506-binding protein 5 (FKBP5) gene, are also involved in HPA functioning and potentially mental health outcomes [49]. A meta-analysis revealed that individuals with specific allele variations for the FKBP5 gene had higher risk of developing certain mental health conditions, such as depression or post-traumatic stress disorder [60].

### 5.2. Quality of Evidence

Genetic inheritance undoubtfully impacts one’s resilience to or risk of developing mental health conditions. However, findings are often conflicting, which could be attributable to differences in study designs (e.g., at what age data collection occurred and type of polymorphism examined). Further, there are a plethora of polymorphisms or allelic variations that could arise, which can make it difficult to pinpoint the true effect of a specific genotype or genetic sequence. Since glucocorticoids (e.g., cortisol) are particularly important in orchestrating various physiological processes [61], it is unsurprising that there is some converging evidence of their role in shaping mental health. Replication of existing research findings is needed. Further, findings of various studies need to be pooled to better understand the associations among genotypes and mental health among individuals exposed to ACEs.

## 6. Epigenetics

Epigenetics refers to interactions that occur between genes and the environment and cause changes in genetic expression without altering the core structure of the DNA sequence. Evidence suggests that epigenetic alterations can occur as early as the prenatal period [5]. Many different mechanisms are described in the context of epigenetics, with DNA methylation often epitomized as a subtype of histone modifications [43,45]. DNA methylation involves the addition of methyl groups to certain DNA sites, which almost always results in repressed expression of the target gene [43,45]. Other histone modifications include phosphorylation, acetylation, and ubiquitination, while other epigenetic mechanisms include noncoding ribonucleic acid and ribonucleic epigenetics [62]; however, due to the prominent role of methylation, we focus on this epigenetic mechanism in this review. Since toxic stressors such as ACEs serve as particularly prominent environmental stimuli, it is likely that ACEs can influence genetic expression through pre- and postnatal methylation mechanisms, highlighting the importance of considering epigenetic mechanisms as proxies for higher or lower risk of developing mental health conditions in the context of ACEs [5].

### 6.1. Effects of Childhood Adversity via Prenatal Epigenetic Mechanisms

Several studies have suggested that offspring of mothers who experience prenatal stress, which is more common among mothers with ACEs [30], are at risk of developing adverse mental health conditions through prenatal DNA methylation [5,63] and, potentially, fetal programming mechanisms [5]. For example, studies conducted in the context of extreme stressors such as the Holocaust showed differential methylation patterns of the FK506 binding protein 5 (FKBP5) gene; compared to controls, parents exhibited methylated FKBP5 genetic patterns while newborns exhibited lower methylation patterns in the same gene [9]. Since the FKBP5 gene helps govern the negative feedback loop of the HPA axis, lower methylation of the gene among newborns is believed to increase resilience in anticipation of similar stressors through epigenetic fetal programming mechanisms which increase FKBP5 expression and decrease HPA functioning [9]. Since mothers’ ACEs have been linked to higher prenatal stress, and it is possible for prenatal stress to negatively impact fetal development through DNA methylation [63], it is important to examine whether mothers’ ACEs may intergenerationally impact their children’s health through interactions between the prenatal environment and genetic expression.

A systematic review conducted by Sosnowski et al. examined if prenatal stress was associated with infants’ (less than 12 months) DNA methylation patterns [63]. In their review, Sosnowski et al. found converging evidence for higher levels of methylation in the nuclear receptor subfamily 3 group C member 1 (NR3C1) gene when mothers reported anxiety, depression, or stress during pregnancy [63]. Higher NR3C1 methylation in infants implies reduced NR3C1 activity, resulting in a decreased capacity to inhibit the HPA axis, which leads to hyperactivity of the stress response system [63]. This in turn is associated with more mental health conditions [23]. However, evidence of the effects of prenatal stress on other candidate genes such as those coding for serotonin and oxytocin was inconclusive [63], warranting caution when considering the effects of prenatal stress on methylation patterns of these other genes. Nevertheless, the consistent findings regarding the association between prenatal stress and methylation of the NR3C1 gene reveal an important target gene that could be acted upon to prevent the development of mental health conditions.

### 6.2. Effects of Childhood Adversity via Postnatal Epigenetic Mechanisms

In addition to the prenatal period, direct experiences of ACEs can affect genetic expression and mental health through epigenetic mechanisms [43]. The evidence of epigenetic mechanisms playing a role in the risk of or resilience to human mental health conditions in the context of ACE exposure is underscored in a systematic review conducted by Parade et al., which focused on associations between childhood maltreatment and DNA methylation [45]. In their review of 100 studies (28 focused on children and 72 on adults), a large body of evidence pointed toward increased DNA methylation patterns among those exposed to ACEs [45]. Studies conducted among children focused predominantly on genes regulating the stress response through glucocorticoid signaling (i.e., NR3C1 (n = 6) and FKBP5 (n = 2)); most studies observed higher and lower methylation patterns of the NR3C1 and FKBP5 genes, respectively, among children exposed to adversity [45]. Further, hypermethylation of the gene encoding for the corticotropin releasing hormone (CRH) was also observed; underexpression of this key hormone due to hypermethylation results in hyperactivity of the HPA stress system [45]. These studies point to epigenetic changes in stress-related genes among children after exposure to adversity, providing compelling evidence for a potential mechanism through which ACEs may result in psychopathology [45].

In contrast, findings from adult studies demonstrate more variability [45]. For example, though most observed increased methylation of the NR3C1 gene, some observed no changes in methylation patterns, and others observed decreased methylation [45]. Likewise, variability was observed among adults for the effects of childhood adversity on FKBP5 methylation patterns [45]. Differences in methylation patterns between children and adults can be attributed to differences in study design, as those conducted among children appear to utilize more rigorous methods to measure DNA methylation patterns that are more closely measured to the timing of the ACE exposure [45]. This is particularly important, as genome-wide association studies conducted among children highlighted the importance of timing of exposure to adversity in relation to methylation patterns, which is more difficult to ascertain through retrospective methods such as those used in adult populations [45]. Other reviews that have examined the association between children’s ACEs and DNA methylation have observed similar findings, with strong associations [36], providing mounting evidence that ACEs negatively impact health through alterations in DNA methylation patterns.

### 6.3. Quality of Evidence

Converging evidence from systematic reviews points to an association between ACEs (both experienced directly or transmitted intergenerationally) and altered DNA methylation patterns among children (but not adults), particularly among genes coding for glucocorticoid receptors, e.g., NR3C1 [36,45,63]. Further, moderate-to-high quality ratings of studies included in the described systematic reviews and strong associations between exposure to ACEs and glucocorticoid receptors patterns indicate higher quality of evidence [36,45,63], with inferences toward causality. Conflicting evidence among other genes, particularly among adults, requires further exploration and more universal data collection methods. The findings to date indicate that ACEs likely impact mental health through epigenetic effects on genes related to glucocorticoid receptors, suggesting important targets for intervention.

## 7. Brain Structure and Function

Brain structure and function are correlated with the functionality and expression of the brain [64]. Since mental health is an embodiment of the health and dynamics of the neural network, it is unsurprising that changes in brain structure and function may be associated with increased resilience to or risk of developing specific types of mental health conditions [6]. In addition to the hypothalamus and pituitary gland, scientists have linked several structures in the brain including the amygdala, hippocampus, and the neocortex, among others, to several mental health conditions through changes in the volume (including changes in gray and white matter), synaptic signaling, and shape of these structures [65]. For example, neuroimaging has elucidated that depressed individuals often have less white and gray matter in many of the aforementioned brain structures, both of which are pivotal in processing information within the brain [66]. Dysregulated activity in critical brain regions that promote functions integral to mental health, such as learning, memory, executive functioning, fear response, and impulse control, can undermine how individuals receive stimuli and regulate their responses [6].

Studies have elucidated that mothers’ prenatal stress (which again is more likely among mothers exposed to ACEs [30]) can adversely affect structural and functional brain development in children, predisposing them to developing mental health conditions [67]. Postnatally, direct experiences of ACEs can affect brain structure and development through synaptic pruning (elimination of neurons and synapses that are not required) or axonal branching (establishment of connections to target brain areas) [28]. Therefore, examining brain structures (aside from the hypothalamus and pituitary gland, which have already been discussed) that are strongly affected by exposure to ACEs is imperative in newborns and developing children as this could support the development of interventions that could enforce more positive brain development outcomes.

### 7.1. Prenatal Effects of ACEs on Brain Structure, Function, and Mental Health

Many studies have examined how mothers’ prenatal stress, which is more likely among mothers exposed to ACEs, is associated with children’s brain structure and function [68,69,70,71,72,73]. Studies using brain imaging have observed lower gray matter density or decreased thickness in various brain regions (e.g., the prefrontal, lateral temporal, and premotor cortices, the medial temporal lobe, the postcentral gyrus, the cerebellum) among individuals exposed to prenatal mental health challenges or stress [68,69,70,71,72,73]. More specific to the amygdala, Qiu et al. reported a statistically significantly greater level of connectivity of the amygdala with various brain regions (e.g., left temporal cortex and insula) among 6-month-old infants whose mothers experienced prenatal depression compared to those whose mothers did not experience prenatal depression, independent from postnatal depression, a neurological pattern that is consistent with that of adults experiencing depression [74] and which has been replicated in other studies [75]. Many of these findings are also replicated in animal models (e.g., rats and nonhuman primates) through purposeful induction of prenatal stress [76]. Though some studies report no changes in certain brain structures when individuals are exposed to prenatal mental health conditions or stress [71,77], there is a substantial body of evidence supporting a link between prenatal mental health or stress and children’s brain structure [68,69,70,71,72,73,78], alluding to the importance of supporting mothers prenatally and promoting healthy brain development.

### 7.2. Adverse Childhood Experiences and Brain Structure and Function

The findings regarding the volume of the amygdala in the context of ACE exposure are conflicting; some studies suggest that exposure to certain ACEs (e.g., those experiencing parental depressive symptoms) results in larger amygdala structures, while others (e.g., severe forms of abuse) result in smaller amygdala structures [28]. In contrast, the findings regarding the functional role of the amygdala in those exposed to ACEs are more uniform, such that individuals exposed to ACEs often have a more active amygdala compared to unaffected individuals [79]. In relation to the hippocampus, Herzog et al. identified several studies that demonstrated a reduction in hippocampal volume among individuals exposed to ACEs compared to unexposed individuals, with evidence of sex-based differences such that males showed more reduction than females [28]. Given that the hippocampus plays a role in inhibiting the HPA axis, a decrease in hippocampal volume is expected to upregulate the HPA axis, increasing one’s risk of developing certain mental health conditions (e.g., post-traumatic stress disorder) [28]. Other brain structures (e.g., different gyri such as the superior frontal gyrus and the middle temporal gyrus) have also been observed to change after exposure to ACEs though these structures are less studied [80]. Overall, research points to the role that ACEs play in shaping the brain. The hippocampus and amygdala are the most studied and could be targeted to help alleviate or prevent mental health conditions. For example, interventions targeting stress reduction (e.g., mindfulness-based interventions) have demonstrated reductions in gray matter density in the amygdala structure, reflecting the neuroplastic nature of the brain [81].

### 7.3. Quality of Evidence

Though some differences exist in terms of how prenatal stress and postnatal ACEs impact infant, adolescent, and adult brains, the findings collectively allude to an adverse impact of exposure to prenatal stress or ACEs on brain development. Moreover, differences in findings can also be attributable to the age at which brain neuroimaging occurred as postnatal exposures are likely to effect changes on brain structure and function [28,80], compounding effects that may emerge from prenatal exposures [68,69,70,71,72,73]. Controlling for important factors such as age at time of imaging, gestational age and birthweight, and mothers’ prenatal and postnatal risky health behaviors (e.g., smoking), alongside postnatal adversities or resiliencies (e.g., supportive parenting) is imperative to ensure that findings are more robustly comparable across studies [76]. Additionally, systematic reviews and meta-analyses that synthesize all existing literature, particularly for those exposed to prenatal stress, could help pool available evidence to provide a more rigorous interpretation of the findings. Lastly, differences in brain structure and function are also attributed to the type of mental health conditions under investigation (e.g., a reduction in amygdala functionality is observed among individuals who also report post-traumatic stress disorder [28]), showing the importance of discussing specific brain regions in respect to different types of mental health conditions. Despite these challenges, existing cohort studies provide some inference of temporality, thus permitting some degree of exposure–outcome inferences. Changes in brain structure and function in the context of prenatal stress and postnatal ACE exposure appears to be a promising mechanism to explain the etiology of mental health conditions among these individuals; brain imaging and developmental screening among individuals exposed to ACEs could help healthcare professionals and affected children and caregivers or adults to work collaboratively to alter brain structure and function given the neuroplastic nature of the brain.

## 8. Oxidative Stress

Adenosine triphosphate (ATP) is an important source of energy for many living organisms, which is produced through various mechanisms including oxidative phosphorylation [82]. Oxidative phosphorylation produces reactive oxygen species (ROS) which are normally neutralized and regulated by antioxidants [82]. Humans have two notable systems involved in regulating and protecting the body from ROS: (1) the antioxidant enzyme system and (2) low-molecular-weight antioxidants [83]. The former and the latter are the primary and secondary lines of defense against ROS, but both utilize antioxidants to eliminate ROS or to repair damaged sites [83]. A major factor in the antioxidant line of defense is the nuclear factor erythroid 2-related factor, a transcription factor, which encodes for antioxidants in most human cells [83]. However, other molecules, such as hydroxy radicals and other redox byproducts, isoprostanes, and glutathione S-transferases, serve as important biomarkers of oxidative stress [84].

At normal levels, ROS are healthy, serving as messengers that help to govern other physiological mechanisms; however, under extreme states of oxidative stress, antioxidants become saturated and ineffective, rendering the body susceptible to ROS-induced damage [82]. For example, high levels of ROS have been associated with protein denaturation, cellular degeneration, and loss of synaptic function [82]. Phospholipids are particularly sensitive to ROS exposure and damage [82]. Since the brain has the second highest lipid content of all structures in the body [85], it is particularly vulnerable to the deleterious effects of ROS saturation [82]. In fact, ROS have been linked to dysregulated neurosynaptic signaling, brain–blood barrier permeability, and brain plasticity, all of which have profound implications for one’s mental health state [82]. Studies have revealed associations between exposure to ROS and neurodegenerative disorders such as Parkinson’s and Alzheimer’s Disease [82,86]. As toxic stress deriving from ACEs has been associated with increased amounts of ROS in cells [7,87], investigating whether ACEs impact mental health through oxidative stress could reveal an important point of intervention that can help to optimize mental health.

### 8.1. Prenatal Effects of Oxidative Stress Subsequent to ACEs

Since mothers with ACEs are at-risk of experiencing dysregulated oxidative stress levels [7,87], some studies have investigated whether ROS during pregnancy are associated with offspring’s mental health in both animal and human models. For example, in a rat model, Scott et al. observed higher levels of oxidative stress in rat mothers who experienced prenatal stress and, consequently, an anxious phenotype in rat offspring along with sex-specific brain changes [88]. Moreover, the use of an antioxidant treatment during pregnancy appeared to prevent these changes [88]. Akhtar et al. also observed greater levels of oxidative stress in fetal brain tissue (using a mouse model) when their mothers were treated with oxidants during the last trimester of pregnancy [89]. Of the few conducted human studies, relationships between mothers’ prenatal oxidative stress levels and their children’s behavioral problems, social-emotional well-being, and/or neurodevelopment have been established [90,91,92,93]. Though the prevalence of ACEs among mothers was not always examined in these studies, it is reasonable to assume that mothers with ACEs could be at higher risk of experiencing prenatal oxidative stress, suggesting a mechanism through which mothers’ ACEs may impact their children’s mental health.

### 8.2. Oxidative Stress in the Postnatal Period: Links to ACEs

In their study, Horn et al. assessed the association between ACEs, oxidative stress biomarkers (F2_t_-isoprostanes), and behavioral problems in children, observing higher levels of oxidative stress, and, in turn, more internalizing problems, among children with four or more ACEs [7]. Fanning et al. observed higher levels of oxidative stress markers among individuals with personality disorders; these individuals also reported more occurrence of childhood abuse and trauma, suggesting the potential role of oxidative stress in the etiology of personality disorders among those exposed to ACEs [94]. More specific to sexual abuse, Atabay et al. observed significantly greater amounts of oxidative stress among exposed adolescent children compared to unexposed children [95], while Şimşek et al. observed no significant difference in the amount of oxidative stress between children exposed and unexposed to sexual abuse [96]. Lastly, Oliveira et al. observed more glutathione S-transferase enzyme activity, an enzyme relevant to the mediation of the oxidative stress mechanism, among children residing in more rural areas with parents of lower income and education (alluding to the potential for more childhood adversity); however, no other associations were noted between oxidative stress biomarkers and parental income or education [97]. These postnatal findings, though only preliminary, point to the potential for postnatal oxidative stress to help explain how ACEs can impact mental health.

### 8.3. Quality of Evidence

Collectively, there is some evidence of oxidative stress serving as a pathway through which ACEs may impact mental health. Though there are few human studies, those that are available typically report significant findings and are conducted using population-based cohorts, providing some evidence for oxidative stress as a potential causal mechanism through which ACEs impact mental health. However, more research is needed on this topic before drawing any strong conclusions regarding the role that oxidative stress plays in the etiology of mental health conditions in the context of ACEs. Further, examination of different oxidative stress biomarkers limits cross-study comparisons, preventing any strong inferences from being drawn. Therefore, more studies of a similar nature are needed to ensure reproducibility. Once more studies are conducted, a meta-analysis could help to synthesize their findings and to better elucidate the role of oxidative stress in the etiology of mental health among those exposed to ACEs.

## 9. Metabolic Profiles

Metabolic profiles characterize the levels of certain metabolites, including those of glucose, lipids, serum proteins, and carbohydrate profiles [8]. The connection between the gut and the brain, or the gut–brain axis, has been described as an important connection to mental health [98]. Since metabolites fuel the brain, and gut-derived chemical messengers (e.g., neurotransmitters) send signals to the brain, a healthy digestive system and gut microbiome have been associated with more optimal central nervous system functioning [99]. In fact, there is growing interest in examining the gut microbiome as a potential risk factor for several mental health conditions, including anxiety, depression, schizophrenia, and autism spectrum disorder [98]. Moreover, metabolic profiles, including those of glucose and lipids, are of interest given their close link to many biological pathways (e.g., inflammatory processes) that impact brain development and mood [8]. Abnormal metabolic patterns (e.g., high glucose) during pregnancy have also been linked to poorer fetal development trajectories, placing children at risk of poorer cognitive development and mental health outcomes [100]. Studies focusing on the effects of ACEs on digestive problems have elucidated an increased odds of diabetes, liver, and digestive disease among individuals exposed to four or more ACEs relative to zero [1], alluding to the idea that ACEs impact physical and potentially mental health through their effect on metabolism and the digestive system.

### 9.1. How Are Metabolic Profiles during Pregnancy Related to Mental Health: A Focus on ACEs

Dysregulation of the digestive system among expecting mothers is thought to impact fetal development due to irregular nutrient and metabolite levels within the fetal environment, which prevents normal growth [101]. Studies have demonstrated a positive association between the number of maternal ACEs and the risk of gestational diabetes, exhibiting a 39% higher risk in some cases [102], suggesting that mothers with ACEs are more likely to have irregular glucose levels during pregnancy. Consequently, high levels of blood glucose during pregnancy places children at higher risk of developing mental health conditions; for example, hyperglycemia during pregnancy has been associated with anxiety, depression, and behavioral disorders in children [101]. Ranchod et al. observed higher rates of weight gain during pregnancy among mothers exposed to ACEs than unexposed mothers [103], and Diesel et al. observed a positive association between childhood trauma scores and excessive weight gain during pregnancy among anxious or depressed women [104]. Irregular weight gain during pregnancy has in turn been associated with mental health conditions in children including attention-deficit hypertensive disorder and autism spectrum disorder [105,106]. Therefore, there are preliminary studies that support the role of metabolic profiles in the etiology of mental health conditions among infants exposed to prenatal stress.

### 9.2. Impacts of Direct Childhood Adversity on Metabolic Profiles

The evidence supporting the association between exposure to ACEs and unhealthy metabolic profiles is more apparent when directly experiencing ACEs. For example, a positive association between exposure to ACEs and unfavorable lipid levels [107,108,109,110,111,112] has been established, which, in turn, has at times been associated with mental health conditions such as depression [113,114] and schizophrenia [112]. Some studies have also illuminated different metabolic outcomes based on the type of ACE experienced. Miller and Lacey revealed that among adults who experienced ACEs: (1) parental separation, divorce, and/or psychological abuse were associated with lower high-density lipoprotein cholesterol, (2) physical neglect was associated with poorer lipid profiles, (3) parental criminal offending was associated with higher triglycerides and glycated hemoglobin, and (4) parental death or substance use were not associated with any metabolic profile [110]. Gut-regulated metabolites related to the glutamate pathway collected through fecal samples have also been correlated with exposure to early adversity [115], and, in fact, have been identified as potential mediators of the association between ACEs and mental health conditions [116]. Collectively, this evidence points to the potential for metabolism and digestive functioning to be pathways through which ACEs impact mental health; however, additional research is required to identify the most relevant metabolites and metabolic profiles.

### 9.3. Quality of Evidence

When taken collectively, metabolites and their role in various physiological processes are important to consider when examining the etiology of mental health conditions in the context of ACEs. Though we know that metabolites play an important role in mental health [98], direct analyses investigating the associations between prenatal stress and diverse metabolites and gut microbiota in children are lacking, preventing any strong inferences from being drawn. However, the evidence that postnatal experience of ACEs may impact metabolic profiles is promising, particularly for lipid and glucose profiles. It does appear, however, that glucose and its glutamate-derived neurotransmitters may play an important role in mental health conditions in the context of ACEs. Given the available evidence, targeting glucose levels during pregnancy among those exposed directly to ACEs may help in preventing the development of certain mental health conditions; however, further research is needed. Further attention should also be paid to examining lipid profiles, particularly as the brain is largely composed of lipids, which are integral to promoting healthy functioning [117].

## 10. Limitations and Implications

Although narrative methods enable a synthesis of the literature, conclusions may be limited by the lack of rigor in systematic searching and data extraction [18]. Literature was selected to address the purpose of the review, so strict inclusion and exclusion criteria were not employed, raising the possibility that relevant studies were missed and reducing reproducibility [18]. However, we included a large compendium of relevant literature, which could help improve understanding of the various physiological mechanisms’ impacts on mental health in the context of ACEs. This narrative review also allowed us to identify knowledge gaps in the current research. Further, we did not critically appraise each individual paper included in this review as this was beyond the scope. However, we described the quality of evidence for each mechanism using the hierarchy of evidence, which considers the various methodologies used in the literature reviewed [19].

A major limitation to the interpretation of the literature is the lack of attention to the type, timing, frequency, and severity of ACE exposure [118,119]. Future research could be more attentive to the impact of these factors on mental health outcomes when interpreting findings. Use of more consistent data collection procedures for each physiological mechanism, preferably with gold standard measures (e.g., venous blood draw for inflammatory markers [120]), is needed in future research to enhance cross-study comparisons and reproducibility. Importantly, though these mechanisms are technically different, they are intrinsically linked through chemical signals and physiological processes, indicating a need to examine systems concomitantly. For example, epigenetic changes can influence how inflammation is regulated and how HPA-related genes are expressed [45]. Future research that examines how all these mechanisms are functioning through the collection of multiple biomarkers could help address which mechanisms are most pronounced for various types of mental health conditions. Longitudinal studies examining relevant biomarkers and mental health over time are also needed. Elucidating how each mechanism may be connected to specific types of mental health conditions can help tailor interventions to reduce and prevent mental health conditions among those exposed to ACEs and increase one’s mental health resilience.

## 11. Concluding Remarks

This review employed narrative synthesis methods to describe how HPA axis functioning, inflammation, genetic inheritance and differential susceptibility, epigenetics, brain structure and function, oxidative stress, and metabolic profiles may help explain the association between ACEs and the development of mental health conditions. For all mechanisms, suitable evidence suggests implication in mental health conditions among those exposed to ACEs (Table 2). The role of inflammation in the etiology of mental health conditions among those exposed to ACEs appears most promising, followed by HPA axis functioning, brain structure and function, genetics, epigenetics, metabolic profiles, and lastly, oxidative stress. Future research should explore interrelationships among these mechanisms and employ longitudinal studies to inform interventions.

## Figures and Tables

**Table 1:** Summary table of genes/genetic sequences affiliated with children’s resiliency in the context of childhood adversity.

Gene/Genetic Sequence	Description and Physiological Role
5-HTTPLR	A serotonin-transporter-linked promoter region on the SLC6A4 gene, affecting the level of genetic expression of the gene and subsequent reuptake of serotonin from the synapse [47].
CRHR1	The corticotropin releasing hormone receptor 1 is a gene that is involved with the release and subsequent binding of corticotropin releasing hormones important in various biological mechanisms, particularly the hypothalamic–pituitary–adrenal axis [48].
FKBP5	FKBP5 proteins are important in downregulating the hypothalamic–pituitary–adrenal axis and are therefore involved in governing the level of cortisol molecules circulating in the body [49].
OXTR	It is a receptor for oxytocin which is important for social and emotional wellbeing, cardiovascular tissue, and labor, serving to reduce stress [50].
BDNF	This neurotransmitter is important in regulating neuron longevity, glucose levels, and metabolism [51].

NOTE: 5-HTTPLR = serotonin transporter promoter polymorphism of SLC6A4; CRHR1 = corticotropin-releasing hormone receptor 1; FKBP5 = FK506-binding protein 5; OXTR = oxytocin receptor gene; BDNF = brain-derived neurotrophic factor.

**Table 2:** Summary of review findings.

Mechanism	Prenatal Evidence	Postnatal Evidence
Hypothalamic–Pituitary–Adrenal (HPA) Axis	- Mothers’ prenatal stress (which is often greater for mothers who experienced adverse childhood experiences (ACEs)) appears to be associated with high levels of cortisol in children postnatally, showing how ACEs may predispose children to developing mental health conditions through hypercortisolism as early as the prenatal period	- For most mental health conditions (e.g., anxiety and depression), individuals often exhibit hypercortisolism when exposed to ACEs - When mental health conditions are absent among those exposed to ACEs, individuals often exhibit hypocortisolism - Hypocortisolism is more likely to be observed with increasing age - Timing and type of ACE exposure appear to largely impact the direction of the state of cortisol - Lack of controlling for prenatal conditions can confound and impact interpretations of postnatal HPA mechanisms
Inflammation	- Consistent evidence that mothers’ prenatal stress (which is often greater for mothers who experienced ACEs) is associated with increased inflammation in children, which has been linked to poorer child mental health - The associations between inflammation and mental health conditions are often described as strong associations	- Consistent evidence that ACEs among children are associated with increased inflammation in children, which is linked to poorer child mental health - Timing, type, frequency, and severity of ACEs could better inform how inflammation impacts certain mental health conditions - The magnitude of studied associations is often described as strong, providing greater evidence for inferences of causality
Genetic Inheritance	- -We focused primarily on outcomes of experiencing direct ACEs as opposed to prenatal stress (as much of the literature regarding prenatal stress if focused on epigenetics)	- Certain genes, such as those related to glucocorticoid receptors, appear to be pivotal in the development of mental health conditions among those exposed to adverse or non-adverse conditions - Often, findings are conflicting, which could be attributed to a lack of consistency in study designs
Epigenetics	- Evidence that prenatal stress (which is often greater for mothers who experienced ACEs) undermines glucocorticoid expression (through DNA methylation of the glucocorticoid receptor), potentially affecting mental health outcomes - Conflicting evidence regarding the associations between DNA methylation in other genes and mental health conditions when exposed to prenatal stress	- Evidence that ACEs impact glucocorticoid expression through DNA methylation, which in turn could explain the development of certain mental health conditions - Conflicting evidence regarding the associations between DNA methylation in other genes and mental health conditions when exposed to ACEs - The magnitude of studied associations is often described as strong, providing greater evidence for inferences of causality
Brain Structure and Function	- Evidence that prenatal stress (which is often greater for mothers who experienced ACEs) is associated with brain structure and function, which in turn are associated with mental health conditions - Lack of meta-analytic analysis prevents pooled estimations of strength and direction of associations in relation to specific brain structures and functions	- Evidence that ACEs are associated with changes in brain structure and function which in turn are associated with different mental health conditions - Certain brain structures have been linked to specific mental health conditions, providing a better understanding of the potential brain basis of mental health conditions - Researchers often fail to control for prenatal conditions and other environmental factors
Oxidative Stress	- Apparent association between prenatal stress (which is often greater for mothers who experienced ACEs) and mental health conditions through oxidative stress - Few human studies have been undertaken; therefore, inferences on the association between prenatal stress and oxidative stress are limited	- Apparent association between ACEs and mental health conditions through oxidative stress - Few human studies have been undertaken so inferences on the association between oxidative stress and ACEs are limited; however, studies in humans do focus on specific types of ACEs, enhancing the strength of interpretation - Research that replicates existing studies are required to better understand how oxidative stress can lead to mental health conditions in the context of exposure to ACEs
Metabolic Processes	- Preliminary evidence that prenatal stress (which is often greater for mothers who experienced ACEs) undermines metabolism, particularly glucose-derived metabolites, which could then potentiate mental health conditions - Lack of direct analyses hinders strong inferences on the role of metabolism and prenatal ACE exposure	- Some evidence that ACEs could impact mental health conditions through metabolic processes, particularly glucose-derived metabolites - Understanding is limited due to a lack of research directly analyzing the associations between ACEs and mental health conditions via metabolic processes - More research is needed to better understand how ACEs may impact other metabolic profiles and, ultimately, mental health conditions

## Data Availability

No new data were created or analyzed in this study. Data sharing is not applicable to this article.
